# Modeling the Effects of Nanopatterned Surfaces on Wetting States of Droplets

**DOI:** 10.1186/s11671-017-2086-9

**Published:** 2017-04-26

**Authors:** Ke Xiao, Yanping Zhao, Gang Ouyang, Xinlei Li

**Affiliations:** 1 MOE Key Laboratory of Laser Life Science & Institute of Laser Life Science, College of Biophotonics, South China Normal University, Guangzhou, 510631 China; 2 Key Laboratory of Low-Dimensional Quantum Structures and Quantum Control of Ministry of Education, Synergetic Innovation Center for Quantum Effects and Applications (SICQEA), Hunan Normal University, Changsha, 410081 China

**Keywords:** Nanopatterned superhydrophobic surface, Thermodynamic method, Wetting state, Free energy

## Abstract

An analytic thermodynamic model has been established to quantitatively investigate the wetting states of droplets on nanopatterned surfaces. Based on the calculations for the free energies of droplets with the Wenzel state and the Cassie-Baxter state, it is found that the size and shape of nanostructured surfaces play crucial roles in wetting states. In detail, for nanohole-patterned surfaces, the deep and thin nanoholes lead to the Cassie-Baxter state, and contrarily, the shallow and thick nanoholes result in the Wenzel state. However, the droplets have the Wenzel state on the patterned surfaces with small height and radii nanopillars and have the Cassie-Baxter state when the height and radii of nanopillars are large. Furthermore, the intuitive phase diagrams of the wetting states of the droplet in the space of surface geometrical parameters are obtained. The theoretical results are in good agreement with the experimental observations and reveal physical mechanisms involved in the effects of nanopatterned surfaces on wetting states, which implies that these studies may provide useful guidance to the conscious design of patterned surfaces to control the wetting states of droplets.

## Background

Hydrophobic materials can be turned into superhydrophobic ones if their surfaces are decorated with micro- or nanocorrugations. Superhydrophobic surfaces have attracted much interest in both fundamental research and industry. This is mainly due to their unique wetting properties and promising technologic application, e.g., in coating, slef-cleaning, and ultra liquid repellent [1–8]. In reality, the superhydrophobicity of lotus leaves is owing to their own hierarchical pillared structure; more specifically, a solid surface can be significantly enhanced by the presence of nano- or micro-scale pillars [9]. On the other hand, the superhydrophobicity also can be influenced by the coulomb repulsion at nanometer-sized contact [10]. A water droplet sitting on a micro-structure surface generally fully wets the substrate topography or is suspended atop the surface topography without penetration, namely, either is the Wenzel (W) [11] state or Cassie-Baxter (CB) [12] state.

Understanding the mechanism of the wetting transition between the W and CB states on nanodecorated surfaces is of essential importance for the design and fabrication of nanostructured surfaces. Much effort has been devoted to this issue in recent years in experiments [13–15] and theories [16–20]. Experimentally, Martines et al. [13] investigated the hydrophilicity, hydrophobicity, and sliding behavior of water droplets on nanoasperities of controlled dimensions and found that the droplet is in the CB state on the pillared surface with large radii and height, but the droplet is in the W state when the pillar radii and height are small. He et al. [14] fabricated a series of micro-rod surfaces with different geometric parameters to study the relationship between micro-rod geometry and wetting state transition and found the similar phenomenon with that observed by Martines et al. Theoretically, based on energy balance, Patankar [18] studied how the surface roughness impacts the wetting state transition of a droplet from a higher energy of the CB state to a lower energy of the W state. Bhushan and Jung [21] demonstrated that hierarchical roughness can result in different superhydrophobic states due to the effects of multiscale roughness on the droplet wetting. In addition, for a pillar-patterned surface, Extrand [22] and Wang and Chen [23] respectively proposed some theoretical criterions to predict wetting states of droplets according the surface geometrical morphology. In spite of much progress in experiments and theories, the effects of the nanopatterned surfaces on wetting states of droplets have not been well understood and there are some fundamental issues in the wetting phenomena on nanopatterned surfaces. For example, the specific relations between the wetting states and geometrical morphology of the patterned surface are unclear, especially for the nano- and micro-holed surfaces. An intuitive phase diagram of wetting states of a droplet in the space of surface geometrical parameters is eagerly expected.

In order to address these problems, in this paper, we took two typical nanodecorated surfaces as an example, i.e., periodic nanohole-patterned surface and nanopillar-patterned surface, respectively. By calculating the free energy difference between the W and CB states, the dependence of wetting behaviors on the periodic nanopatterned geometrical parameters is studied. In detail, we found that for nanohole-patterned surfaces, they tend to be in Cassie-Baxter state as the hole is higher and thinner and tend to be in the Wenzel state as the hole is shorter and thicker. However, for nanopillar-patterned surfaces, the short and thin nanoholes lead to the Wenzel state, and contrarily, the long and thick nanoholes result in the Cassie-Baxter state. In addition, we discussed in detail the intrinsic mechanism of the wetting states of droplets on different patterned surfaces. Furthermore, the intuitive phase diagrams of wetting states of the droplet on different patterned surfaces are obtained. In this way, the phase diagrams offer a simple method to evaluate the wetting states of droplets on nanopatterned surfaces.

## Methods

**Fig. 1:**
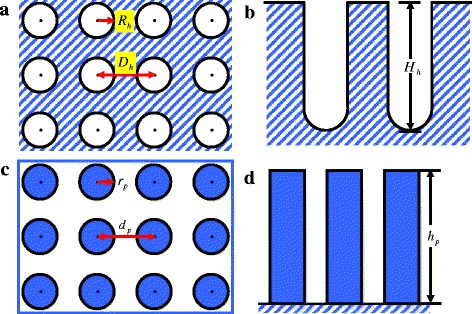
Schematic depiction of a surface decorated with a regular array of nanoholes and nanopillars. Plan view of a surface textured with a regular array of nanoholes of radii, *R*_h_, and center-to-center spacing, *D*_h_ (**a**). Side view of a surface textured with a regular array of nanoholes of height, *H*_h_ (**b**). Plan view: the radii and center-to-center spacing of nanopillars are *r*_p_ and *d*_p_, respectively (**c**). Side view: the height of nanopillars is *h*_p_ (**d**)

To study the wetting behaviors of a droplet on nanopatterned surfaces, we consider the wetting of a water droplet deposited on two typical nanopatterned surfaces, in which the solid surfaces are nanodecorated with a regular array of holes and pillars, and the top view of the periodically distributed nanopatterned surfaces are shown in Fig. 1a, c. Figure 1a shows the plan view of the nanohole-patterned surface with hole radii *R*_h_ and spacing *D*_h_. In addition, the bottoms of the nanoholes are hemispherical in shape (see Fig. 1b). The lateral cross-section of nanoholes with depth *H*_h_ is illustrated in Fig. 1b. Also, Fig. 1c, d depict the plan view and the side view of cylindrical pillars, in which *r*_p_, *h*_p_, and *d*_p_ are the radii of the pillars, the height of the pillars, and the spacing between the pillars, respectively. Geometrically, the nanohole and nanopillar densities are given by ${\rho}_{\mathrm{h}}=1/{D}_{\mathrm{h}}^2$ and ${\rho}_{\mathrm{p}}=1/{d}_{\mathrm{p}}^2$.

**Fig. 2:**
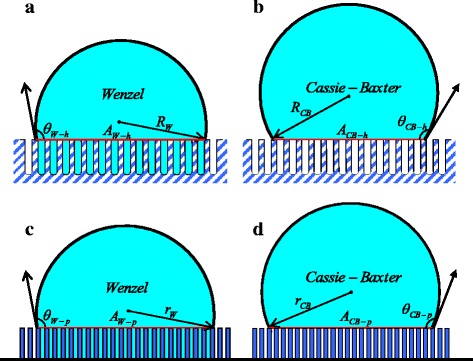
Schematic illustrations of wetting states of a droplet on a nanohole-patterned surface with the W state (**a**) and the CB state (**b**) and on a nanopillar-patterned surface with the W state (**c**) and the CB state (**d**)

Generally, for a system of a droplet placed on a nanopatterned surface, the final state is either the W state or the CB state. The cross-sections of the W and CB states, for a water droplet sitting on the solid surfaces textured by a regular array of nanoholes and nanopillars, are shown in Fig. 2. Based on thermodynamics, the free energy of a droplet on a solid substrate can be calculated by *E* = *γ*_lv_ *S*_lv_ + (*γ*_ls_ − *γ*_sv_)*S*_ls_ [24–27], where *γ*_lv_, *γ*_ls_, and *γ*_sv_ are the liquid-vapor, liquid-solid, and solid-vapor interface energies, respectively, and are satisfied with Young’s equation cos*θ*_0_ = (*γ*_sv_ − *γ*_ls_)/*γ*_lv_ [28–30], and *S*_lv_ and *S*_ls_ are the areas of the liquid-vapor interface and the liquid-solid interface, respectively. Additionally, we have noticed that there are some theoretical reports [31, 32] related to the size-dependent surface energy and interface energy. However, considering the size of droplets is relatively large, we neglect the size dependence of the surface energy and the interface energy in our model. By employing thermodynamics, we will theoretically study the relationship between the nanopatterned surface morphology and the wetting behaviors. The two typical analytical models are detail discussed as follows.

### Analytical Model of Nanohole-Patterned Surface

When the water droplet is placed on a periodically patterned nanohole substrate and is the W state (see Fig. 2a), the corresponding free energy is expressed as

1 $$ {E}_{\mathrm{W}\hbox{-} \mathrm{h}}={\gamma}_{\mathrm{lv}}{S}_{\mathrm{lv},\mathrm{W}\hbox{-} \mathrm{h}}+\left({\gamma}_{\mathrm{ls}}-{\gamma}_{\mathrm{sv}}\right){S}_{\mathrm{ls},\mathrm{W}\hbox{-} \mathrm{h}} $$

In the system under study, we consider the droplet to be big enough that the volume of each nanohole is negligible when compared to the droplet volume. The boundary effect, as a result, can be ignored in the contribution of free energy. In Eq. (1), the geometric parameters are that

2 $$ {S}_{\mathrm{lv},\mathrm{W}\hbox{-} \mathrm{h}}=2\pi {R}_{\mathrm{W}}^2\left(1- \cos {\theta}_{\mathrm{W}\hbox{-} \mathrm{h}}\right) $$

3 $$ {S}_{\mathrm{ls},\mathrm{W}\hbox{-} \mathrm{h}}={A}_{\mathrm{W}\hbox{-} \mathrm{h}}\left(1-{\rho}_{\mathrm{h}}\pi {R}_{\mathrm{h}}^2+2{\rho}_{\mathrm{h}}\pi {R}_{\mathrm{h}}{H}_{\mathrm{h}}\right) $$

4 $$ {V}_{\mathrm{W}\hbox{-} \mathrm{h}}=\frac{1}{3}\pi {R}_{\mathrm{W}}^3{\left(1- \cos {\theta}_{\mathrm{W}\hbox{-} \mathrm{h}}\right)}^2\left(2+ \cos {\theta}_{\mathrm{W}\hbox{-} \mathrm{h}}\right)+{A}_{\mathrm{W}\hbox{-} \mathrm{h}}{\rho}_{\mathrm{h}}\left(\pi {R}_{\mathrm{h}}^2{H}_{\mathrm{h}}-\frac{1}{3}\pi {R}_{\mathrm{h}}^3\right) $$

where *S*_lv,W-h_ and *S*_ls,W-h_ represent the liquid-vapor interface area and the liquid-solid interface area, ${A}_{\mathrm{W}\hbox{-} \mathrm{h}}=\pi {R}_{\mathrm{W}}^2{ \sin}^2{\theta}_{\mathrm{W}\hbox{-} \mathrm{h}}$ denotes the projected droplet base surface area, and *R*_W_ and *V*_W-h_ are the droplet radii and volume in the Wenzel model, respectively. By substituting Eqs. (2), (3), and Young’s equation into Eq. (1), thus, the free energy of the W state of a droplet on the nanohole-patterned surface can be rewritten as

5 $$ {E}_{\mathrm{W}\hbox{-} \mathrm{h}}={\gamma}_{\mathrm{lv}}\left[2\pi {R}_{\mathrm{W}}^2\left(1- \cos {\theta}_{\mathrm{W}\hbox{-} \mathrm{h}}\right)-\pi {R}_{\mathrm{W}}^2{ \sin}^2{\theta}_{\mathrm{W}\hbox{-} \mathrm{h}} \cos {\theta}_0\left(1-{\rho}_{\mathrm{h}}\pi {R}_{\mathrm{h}}^2+2{\rho}_{\mathrm{h}}\pi {R}_{\mathrm{h}}{H}_{\mathrm{h}}\right)\right] $$

Similarly, for the system of a water droplet suspended above the nanohole-patterned surface, i.e., the CB state (see Fig. 2b), the relevant free energy can be expressed as

6 $$ {E}_{\mathrm{CB}\hbox{-} \mathrm{h}}={\gamma}_{\mathrm{lv}}{S}_{\mathrm{lv},\mathrm{CB}\hbox{-} \mathrm{h}}+\left({\gamma}_{\mathrm{ls}}-{\gamma}_{\mathrm{sv}}\right){S}_{\mathrm{ls},\mathrm{CB}\hbox{-} \mathrm{h}} $$

According to Fig. 2b, we have

7 $$ {S}_{\mathrm{lv},\mathrm{CB}\hbox{-} \mathrm{h}}=2\pi {R}_{\mathrm{CB}}^2\left(1- \cos {\theta}_{\mathrm{CB}\hbox{-} \mathrm{h}}\right)+{A}_{\mathrm{CB}\hbox{-} \mathrm{h}}{\rho}_{\mathrm{h}}\pi {R}_{\mathrm{h}}^2 $$

8 $$ {S}_{\mathrm{ls},\mathrm{CB}\hbox{-} \mathrm{h}}={A}_{\mathrm{CB}\hbox{-} \mathrm{h}}\left(1-{\rho}_{\mathrm{h}}\pi {R}_{\mathrm{h}}^2\right) $$

9 $$ {V}_{\mathrm{CB}\hbox{-} \mathrm{h}}=\frac{1}{3}\pi {R}_{\mathrm{CB}}^3{\left(1- \cos {\theta}_{\mathrm{CB}\hbox{-} \mathrm{h}}\right)}^2\left(2+ \cos {\theta}_{\mathrm{CB}\hbox{-} \mathrm{h}}\right) $$

where *S*_lv,CB-h_ and *S*_ls,CB-h_ refer to the liquid-vapor interface area and the liquid-solid interface area, respectively, ${A}_{\mathrm{CB}\hbox{-} \mathrm{h}}=\pi {R}_{\mathrm{CB}}^2{ \sin}^2{\theta}_{\mathrm{CB}\hbox{-} \mathrm{h}}$ denotes the projected droplet base surface area, and *R*_CB_ and *V*_CB-h_ are the droplet radii and volume in the Cassie-Baxter model, respectively. When combining Eqs. (7), (8), and Young’s equation, in this way, the free energy of the CB state of a droplet on a nanohole-patterned surface is

10 $$ {E}_{\mathrm{CB}\hbox{-} \mathrm{h}}={\gamma}_{\mathrm{lv}}\left[2\pi {R}_{\mathrm{CB}}^2\left(1- \cos {\theta}_{\mathrm{CB}\hbox{-} \mathrm{h}}\right)+{\rho}_{\mathrm{h}}{\pi}^2{R}_{\mathrm{CB}}^2{R}_{\mathrm{h}}^2{ \sin}^2{\theta}_{\mathrm{CB}\hbox{-} \mathrm{h}}-{A}_{\mathrm{CB}\hbox{-} \mathrm{h}} \cos {\theta}_0\left(1-{\rho}_{\mathrm{h}}\pi {R}_{\mathrm{h}}^2\right)\right] $$

With the aim of investigating the influence of geometrical morphology parameters of nanohole-patterned surfaces on wetting states, the free energy of the W and CB states are calculated according to Eqs. (5) and (10). Furthermore, in order to obtain a phase diagram of wetting states of a water droplet on a nanohole-patterned surface, the free energy difference between the W and CB states are calculated. Here, we define the free energy difference between the W and CB states as Δ*E* = *E*_W_ − *E*_CB_. Thus, by combining Eqs. (5) and (10), the free energy difference can be obtained as Δ*E*_h_ = *E*_W-h_ − *E*_CB-h_ for a nanohole-patterned analytical model. When Δ*E*_h_ > 0, i.e., *E*_CB-h_ < *E*_W-h_, it indicates that the CB state will be the final equilibrium state. When Δ*E*_h_ < 0, it implies that the W state is more stable. Accordingly, when Δ*E*_h_ = 0 the boundary of the phase diagram of wetting states is attained to separate the Wenzel state and the Cassie-Baxter state.

### Analytical Model of Nanopillar-Patterned Surface

Figure 2c, d show a water droplet being put directly on a nanopillar-patterned surface and taking the W state or the CB state. Analogous to the previous section, the same derivation procedure is employed to the nanopillar-patterned analytical model. For the wetting states as shown in Fig. 2c, d, we have

11 $$ {S}_{\mathrm{lv},\mathrm{W}\hbox{-} \mathrm{p}}=2\pi {r}_{\mathrm{W}}^2\left(1- \cos {\theta}_{\mathrm{W}\hbox{-} \mathrm{p}}\right) $$

12 $$ {S}_{\mathrm{ls},\mathrm{W}\hbox{-} \mathrm{p}}={A}_{\mathrm{W}\hbox{-} \mathrm{p}}\left(1+2{\rho}_{\mathrm{p}}\pi {r}_{\mathrm{p}}{h}_{\mathrm{p}}\right) $$

13 $$ {V}_{\mathrm{W}\hbox{-} \mathrm{p}}=\frac{1}{3}\pi {r}_{\mathrm{W}}^3{\left(1- \cos {\theta}_{\mathrm{W}\hbox{-} \mathrm{p}}\right)}^2\left(2+ \cos {\theta}_{\mathrm{W}\hbox{-} \mathrm{p}}\right)+{A}_{\mathrm{W}\hbox{-} \mathrm{p}}{h}_{\mathrm{p}}\left(1-{\rho}_{\mathrm{p}}\pi {r}_{\mathrm{p}}^2\right) $$

14 $$ {S}_{\mathrm{lv},\mathrm{CB}\hbox{-} \mathrm{p}}=2\pi {r}_{\mathrm{CB}}^2\left(1- \cos {\theta}_{\mathrm{CB}\hbox{-} \mathrm{p}}\right)+{A}_{\mathrm{CB}\hbox{-} \mathrm{p}}\left(1-{\rho}_{\mathrm{p}}\pi {r}_{\mathrm{p}}^2\right) $$

15 $$ {S}_{\mathrm{ls},\mathrm{CB}\hbox{-} \mathrm{p}}={A}_{\mathrm{CB}\hbox{-} \mathrm{p}}{\rho}_{\mathrm{p}}\pi {r}_{\mathrm{p}}^2 $$

16 $$ {V}_{\mathrm{CB}\hbox{-} \mathrm{p}}=\frac{1}{3}\pi {r}_{\mathrm{CB}}^3{\left(1- \cos {\theta}_{\mathrm{CB}\hbox{-} \mathrm{p}}\right)}^2\left(2+ \cos {\theta}_{\mathrm{CB}\hbox{-} \mathrm{p}}\right) $$

where *S*_lv,W-p_, *S*_lv,CB-p_, *S*_ls,W-p_, and *S*_ls,CB-p_ represent the liquid-vapor interface area and the liquid-solid interface area, respectively; ${A}_{\mathrm{W}\hbox{-} \mathrm{p}}=\pi {r}_{\mathrm{W}}^2{ \sin}^2{\theta}_{\mathrm{W}\hbox{-} \mathrm{p}}$ and ${A}_{\mathrm{CB}\hbox{-} \mathrm{p}}=\pi {r}_{\mathrm{CB}}^2{ \sin}^2{\theta}_{\mathrm{CB}\hbox{-} \mathrm{p}}$ denote the projected droplet base surface area; and *r*_W_, *r*_CB_, *V*_W-p_, and *V*_CB-p_ are the droplet radii and volume in the Wenzel and Cassie-Baxter models, respectively. Therefore, the related free energy of the W and CB states on the nanopillar-patterned surface can be calculated as follows

17 $$ {E}_{\mathrm{W}\hbox{-} \mathrm{p}}={\gamma}_{\mathrm{lv}}\left[2\pi {r}_{\mathrm{W}}^2\left(1- \cos {\theta}_{\mathrm{W}\hbox{-} \mathrm{p}}\right)-\pi {r}_{\mathrm{W}}^2{ \sin}^2{\theta}_{\mathrm{W}\hbox{-} \mathrm{p}} \cos {\theta}_0\left(1+2{\rho}_{\mathrm{p}}\pi {r}_{\mathrm{p}}{h}_{\mathrm{p}}\right)\right] $$

18 $$ {E}_{\mathrm{CB}\hbox{-} \mathrm{p}}={\gamma}_{\mathrm{lv}}\left[2\pi {r}_{\mathrm{CB}}^2\left(1- \cos {\theta}_{\mathrm{CB}\hbox{-} \mathrm{p}}\right)+\pi {r}_{\mathrm{CB}}^2{ \sin}^2{\theta}_{\mathrm{CB}\hbox{-} \mathrm{p}}\left(1-{\rho}_{\mathrm{p}}\pi {r}_{\mathrm{p}}^2-{\rho}_{\mathrm{p}}\pi {r}_{\mathrm{p}}^2 \cos {\theta}_0\right)\right] $$

to better understand the effects of nanopillar-patterned surface factors on droplet wetting behaviors and obtain a certain phase diagram of wetting states of a water droplet on a nanopillar-patterned surface. A similar strategy as the previous section is applied to investigate the intrinsic mechanism of wetting states on a nanopillar-patterned surface in this part. By combining Eqs. (17) and (18), the free energy difference is given by ∆*E*_p_ = *E*_W-p_ − *E*_CB-p_. When ∆*E*_p_ > 0, it represents CB state. When ∆*E*_p_ < 0, it represents the W state. According to ∆*E*_p_ = 0, the boundary of the phase diagram of wetting states is obtained to separate the Wenzel state and the Cassie-Baxter state as well.

Here, we consider that the droplets’ volumes have the same value and are constant. The contact angles *θ*_W_ and *θ*_CB_ are expressed by cos*θ*_W_ = *r*cos*θ*_0_ and cos*θ*_CB_ = *f*cos*θ*_0_ − (1 − *f*), respectively, where *r* and *f* are the respective Wenzel roughness factor and Cassie-Baxter roughness factor. It is notable that there might be energy barriers when the transition between the CB and W states happens and the lower energy state will occur as long as the energy barriers are overcome. However, the energy barriers are usually rather low; in some instances, the energy barriers can be overcome with the help of sufficient conditions such as vibration, enough pressure, and extra field. Accordingly, we consider that the lower energy state is more stable in our analytical models.

## Results and Discussion

### Wetting States on the Nanohole-Patterned Surface

**Fig. 3:**
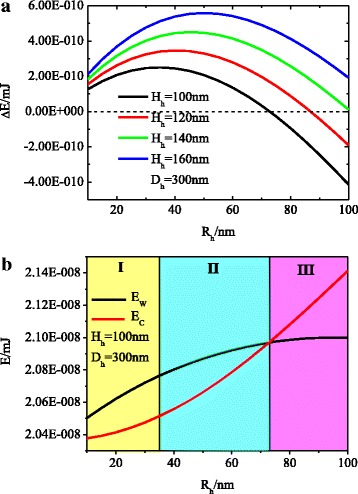
**a** The free energy difference between the W and CB states of a water droplet on nanohole-patterned surfaces when the nanohole depth and spacing have uniform values and varying feature sizes *R*_h_. **b** The individual free energy profiles of the W state and the CB state for the *black line* in (**a**)

Using Eqs. (5) and (10), we can calculate the free energy difference between the W and CB states of a water droplet on a nanohole-patterned surface. In the calculation, we take a water droplet on a nanohole silicon substrate as an example, in which *γ*_lv_ = 73 mJ/m^2^ [33], *θ*_0_ = 112° [34], and *V*_droplet_ = 5.0 × 10^11^ nm^3^. As shown in Fig. 3a, when the nanohole depth and spacing have a uniform value, the water droplet is in the CB state (∆*E*_h_ > 0) as the nanohole radii is smaller than a critical value and in the W state (∆*E*_h_ < 0) when the nanohole radii is larger than the critical value. Also, the critical radii value increases with the increase of nanohole depth, which means that increasing the depth and decreasing the radii of the nanohole will lead to the CB state, and vice versa, it will result in the W state. The reason for the wetting state change is that the free energy is affected by the liquid-vapor interface energy and the liquid-solid interface energy. When a droplet is deposited on a hole-patterned surface, the liquid-solid interface area under the droplet in the W state is larger than the CB state and the liquid-vapor interface area under the droplet in the W state (it is in fact zero) is less than the CB state, which causes the liquid-solid interface energy of the W state to be larger than that of the CB state and the liquid-vapor interface energy of the W state to be smaller than that of the CB state. For deep and thin nanoholes, the liquid-solid interface energy difference between the W and CB states is larger than the liquid-vapor interface energy difference between the CB and W states, which makes the total free energy of the W state larger than the CB state, so in this situation, the CB state is more stable. On the contrary, the W state is more stable for shallow and thick nanoholes. This is because the liquid-solid interface energy difference between the W and CB states is smaller than the liquid-vapor interface energy difference between the CB and W states. From Fig. 3a, we also note that the free energy difference between the W and CB states will ascend to a maximum value and then descend before the radii reach a critical value. To explain the variation tendency of the free energy difference, we have calculated the free energy of the W and CB states individually in Fig. 3b. Regions I and II reflect that the free energy of the W state is greater than the CB state, which indicates that the CB state is stable. However, the increase rates of the free energy of the W state is larger than the CB state in region I and smaller than the CB state in region II, which for the free energy difference renders an ascent first and then a descent. Region III implies that the free energy of the CB state is greater than the W state, which demonstrates that the W state is stable.

**Fig. 4:**
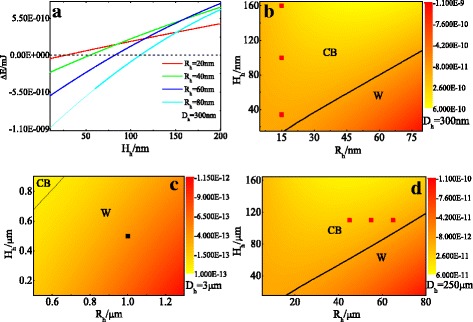
The free energy difference between the W and CB states as the function of hole height *H*_h_ under different nanohole radii (**a**). **b**–**d** are phase diagrams of wetting states of the water droplet on hole-patterned surfaces. In each *panel*, the inserted *square dots* represent experimental measurements compared with our model and the *black line* delineates the division between the W and CB states. Hole spacing of center-to-center *D*_h_ = 300 nm, experimental data from Ref. 35 (**b**); *D*_h_ = 3 nm, experimental data from Ref. 36 (**c**); *D*_h_ = 250 nm, experimental data from Ref. 37 (**d**)

We also calculated that the free energy difference between the W and CB states under the circumstance of nanohole radii and spacing is under a certain value. The results in Fig. 4a clearly show that there exists a threshold depth value to separate the W and CB states. The W state is more stable than the CB state (∆*E*_h_ < 0) as the nanohole depth is smaller than the threshold value, and the CB state will be the final equilibrium state (∆*E*_h_ > 0) as the nanohole depth grows larger than the threshold value. To comprehensively study, moreover, the effect of nanohole radii and depth on wetting states of droplets on nanohole-patterned surfaces, we obtained a phase diagram of the wetting states of a water droplet on the nanohole-patterned surface from the analysis of the free energy difference between the W and CB states as the function of nanohole depth and radii. Figure 4b shows a *H*_h_ − *R*_h_ phase diagram for the free energy difference between the W and CB states of a water droplet on a nanohole-patterned surface with the nanohole spacing as 300 nm. We note that there is a black line to separate the W and CB states which represents that the values of the free energy difference are equal to zero. It is found that ∆*E*_h_ > 0 when the value of *H*_h_ and *R*_h_ is located above the black line, i.e., the CB state. And ∆*E*_h_ < 0 when the value of *H*_h_ and *R*_h_ is located below the black line, i.e., the W state. At this point, it is necessary and worthwhile to compare the results presented in this work with the relevant experimental results. The experimental observations from Checco et al. [35] reported that for a droplet on a nanohole’s silicon surface with the nanohole radius about 15 nm and the nanohole height 34, 100, and 160 nm, the water droplet is in the CB state. As shown by the red dots in Fig. 4b, the wetting states of the experiment data represented are located in the corresponding zones that the theoretical model predicted. Furthermore, we apply our analytical model to micro-scale structured surfaces to further verify our theoretical predictions match the experimental observations well. Figure 4c, d show a *H*_h_ − *R*_h_ phase diagram for the free energy difference between the W and CB states of a water droplet on micro-hole-patterned silicon surfaces with the hole spacing of 3 and 250 μm, respectively. Data from Bico et al. [36] found that the water droplet is in the Wenzel state as the hole radii and height are 1 and 0.5 μm, which is the black dot located in the W region in Fig. 4c. In another study, Jopp et al. [37] investigated the wetting behavior of water droplets on periodically hydrophobic micro-texture structured surfaces, and their experiment results reported that for micro-structure pore surfaces with a center-to-center distance of 250 μm, and the depth of each pore at 110 μm, when the pore radii are 45, 55, 65, and 75 μm, respectively, the water droplets are in the CB state. The red dots in the CB regime in Fig. 4d are the corresponding data, and these experimental observations are consistent with our theoretical predictions.

In summation, the theoretical results are in good agreement with the experimental observations. Figure 4b displays a phase diagram of the W and CB states of a water droplet on a nanohole-patterned surface, which can clarify the interrelated effects of the radius and the height of the nanohole on wetting states. The phase diagram clearly shows the relationship between the geometrical morphology parameters of a nanopatterned surface and the wetting states. Therefore, Fig. 4b is an explicit wetting state phase diagram for the nanohole-patterned analytical model and is applicable for predicting the initial wetting state under the circumstance of a water droplet sitting on different sizes of nanohole-patterned surfaces.

### Wetting States on Nanopillar-Patterned Surface

**Fig. 5:**
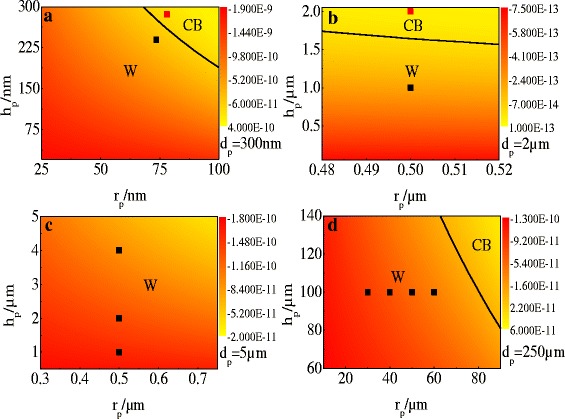
Phase diagram of wetting states of the water droplet on pillar-patterned surfaces. In each *panel*, *square dots* represent experimental results and the *black line* delineates the W-CB boundary. Pillar spacing *d*_p_ = 300 nm, experimental results from Ref. 13 (**a**); *d*_p_ = 2 nm, experimental results from Ref. 38 (**b**); *d*_p_ = 5 nm, experimental results from Ref. 38 (**c**); *d*_p_ = 250 nm, experimental results from Ref. 37 (**d**)

It is also interesting to investigate the wetting behaviors of water droplets on nanopillar-patterned surfaces. In order to further systematically elucidate the dependence of wetting states on nanopillar radii and height, we first calculated the free energy difference between the W and CB states of a water droplet on a nanopillar-patterned surface, with the droplet having the same volume as the previous section. We obtained, therefore, a W-CB phase diagram for a water droplet on a nanopillar-patterned surface while the pillar spacing is 300 nm. As illustrated in Fig. 5a, the W and CB states were separated by a black line which indicates that the values of free energy are zero. It represents ∆*E*_p_ > 0 when the values of *h*_p_ and *r*_p_ are located above the black line, i.e., the CB state. It indicates ∆*E*_p_ < 0 when the values of *h*_p_ and *r*_p_ are located below the black line, i.e., the W state. We note that, form Fig. 5a, as the nanopillar spacing have a uniform value, the wetting state is the CB state, which is more stable than the W state while both the pillar height and radii are larger than the critical values. The W state is more stable while the pillar height and radii are smaller than the critical values. The reason that causes the change of wetting states is mainly because the variation of *h*_p_, *r*_p_, and *d*_p_ will cause the change of the liquid-solid interface energy and the liquid-vapor interface energy. In short, Fig. 5a illustrates that the increase of the pillar height and radius will render to the CB state, while the decrease of the pillar height and radius will lead to the W state. Comparing the results of wetting states on the nanopillar-patterned surface with those on the nanohole-patterned surface, we can find that the thick pillars lead to the CB state, but the CB state on the holed surface corresponds to thin holes. In fact, the physical mechanisms of the different wetting states on the two nanopatterned surfaces are identical. For pillar-patterned surface, thick pillars represent the small space among pillars and the effects of the small space are similar with those of thin holes. Therefore, in these cases, both thick pillars and thin holes result in the CB state.

Finally, to further validate our analytical model, we compare our model predictions with several relevant experimental studies of the wetting properties of water droplets on pillar-patterned silicon surfaces. Martines et al. [13] experimentally investigated the hydrophobicity behavior of water droplets on nanoasperities with fixed center-to-center pitch *d*_p_ = 300 nm and various pillar diameters and heights. They observed that the droplet is in the CB state when the pillar radii and height are 78 and 286 nm, respectively (the red dot in Fig. 5a), and the droplet is in the W state when the pillar radii and height are 73.5 and 239 nm, respectively (the black dot in Fig. 5a), which are located in the corresponding regions that were theoretically predicted. Next, we use the experimental data in micro-scale to confirm our model. Fürstner and co-workers [38] studied the wetting and self-cleaning properties of water droplets on three types of artificial superhydrophobic surfaces with different micro-structure geometries. The experimental results reported that the wetting model is the W state when the geometric parameters are *r*_p_ = 0.5 μm, *h*_p_ = 1.0 μm, and *d*_p_ = 2.0 μm (the black dot in Fig. 5b) and the wetting model is the CB state when the geometric parameters are *r*_p_ = 0.5 μm, *h*_p_ = 2.0 μm, and *d*_p_ = 2.0 μm (the red dot in Fig. 5b). They also found that as *r*_p_ = 0.5 μm, *d*_p_ = 5.0 μm, and *h*_p_ = 1.0, 2.0, and 4.0 μm (the black dots in Fig. 5c, respectively), the wetting models are the W state. In the end, we compare the theoretical results with the experimental results measured by Jopp et al. [37] for water droplets on micro-texture structured surfaces when the pillar height and spacing distance are 250 and 110 μm, respectively; the water droplets are in the W state when the pillar radius is 30, 40, 50, and 60 μm (the black dots in Fig. 5d, respectively). The theoretical results predicted by our model are in excellent agreement with those results observed by experiments. Moreover, the model results can qualitatively explain some experiment results. For instance, He et al. [14, 15] found that increasing the height and decreasing the space of micro-rods may result in the Cassie-Baxter wetting state, while decreasing the height and increasing the space may result in the Wenzel wetting state. The comparison between the related experiment data and the prediction of the theoretical model in this paper are well matched, and the theoretical results’ variation tendency agrees well with the experimental results, which validate our theoretical model.

The above discussions suggest that the droplet wetting behaviors on a nanopillar surface are determined by the surface geometric structure, including nanopillar radius, height, and spacing between the nanopillars. Figure 5a shows a phase diagram of the wetting states on the nanopillar-patterned surface, and it explicitly expresses the relationship between the geometry of the nanopillar-patterned surface and the corresponding wetting state. Therefore, with the help of the wetting state phase diagram, it would be very easy to obtain the initial equilibrium wetting state when a droplet is sitting on a nanopatterned surface by knowing the size of the surface nanostructure.

## Conclusions

In conclusion, we applied the thermodynamic method to study the wetting states of a water droplet on the two typical nanopatterned surfaces. We calculated the free energy difference between the W and CB states. Analyzing the free energy difference, we found that the wetting states of water droplets on nanopatterned surfaces are sensitive to the geometrical morphology and the intrinsic mechanism of wetting states which depends on the periodic nanopatterned geometrical parameters is elucidated in detail. To systematically understand the dependence of wetting states on the geometrical parameters of nanopatterned surfaces, two phase diagrams of the W and CB states for water droplets on nanopatterned surfaces are given, which would be quite useful in predicting the initial wetting state. The theoretical results agree well with the reported experimental results; we hope that the results can provide some useful guidance to the design and fabrication of nanopatterned surfaces with certain wetting characteristics. In this work, we focused on the phase diagrams of water droplets on the nanohole and nanopillar-patterned surfaces with a periodic square lattice distribution. Nonetheless, the thermodynamic method is general, and it can be applied to other nanopatterned surfaces and obtain the corresponding phase diagrams as well.
